# Unlocking the Potential: Angiotensin Receptor Neprilysin and Sodium Glucose Co-Transporter 2 Inhibitors for Right Ventricle Dysfunction in Heart Failure

**DOI:** 10.3390/medicina60071112

**Published:** 2024-07-09

**Authors:** Bibhuti B. Das

**Affiliations:** Heart Failure and Transplant Program, Department of Pediatrics, University of Mississippi Medical Center, Jackson, MS 39216, USA; bdas@umc.edu

**Keywords:** ARNI, SGLT2i, heart failure, right ventricle, left ventricle

## Abstract

This review article examines the mechanism of action of Angiotensin Receptor–Neprilysin Inhibitors (ARNIs) and Sodium–Glucose Co-Transporter 2 Inhibitors (SGLT2is) in managing chronic right ventricular (RV) dysfunction. Despite advancements in heart failure (HF) treatment, RV dysfunction remains a significant contributor to morbidity and mortality. This article explores the The article explores the impact of ARNIs and SGLT2is on RV function based on clinical and preclinical evidence, and the potential benefits of combined therapy. It highlights the need for further research to optimize patient outcomes and suggests that RV function should be considered in future clinical trials as part of risk stratification for HF therapies. This review underscores the importance of the early initiation of ARNIs and SGLT2is as per guideline-directed medical therapy for eligible HFrEF and HFpEF patients to improve co-existing RV dysfunction.

## 1. Introduction

The AHA/ACC/ESC guidelines endorse a quadruple combination therapy, including an angiotensin receptor–neprilysin inhibitor (ARNI), a beta-blocker (BB), a mineralocorticoid receptor antagonist (MRA), and a sodium–glucose co-transporter 2 inhibitor (SGLT2i), for treating heart failure (HF) in adults [1,2]. Despite the remarkable advances in pharmacological therapies, 9.1% of the total deaths in the US were due to HF in 2021 [3]. RV dysfunction remains a significant contributor to morbidity and mortality [4]. Historically, right ventricular (RV) dysfunction has been recognized as part of the HF clinical syndrome, yet it is often overlooked in the risk stratification for HF therapy. However, the advancements in invasive and non-invasive evaluations, combined with clinical and preclinical evidence, have advanced the current understanding of RV pathophysiology across the HF spectrum and reinforced the RV’s centrality in contributing to clinical outcomes. The latest universal definition of HF necessitates the inclusion of symptoms and/or signs linked to cardiac abnormalities and evidence of systemic or pulmonary congestion [5].

RV dysfunction arises from primary myocardial disease, volume overload, pressure overload, and congenital heart disease (CHD) which included RV as systemic ventricle (sRV) that maintain cardiac output [6]. Its relevance to the clinical syndrome of HF is well documented [7,8]. ARNIs and SGLT2is have become essential components of the guideline-directed medical therapy (GDMT) for HF across various ejection fractions (EF), including HFrEF, HFmEF, HFpEF, and HFimpEF [1,2]. While the impact of quadruple HF therapy on left ventricular (LV) function is well established, its effects on RV dysfunction are less well understood. The current GDMT does not explicitly address RV dysfunctioin occuring alongside HFrEF and HFpEF [9,10]. This review synthesizes the clinical trials and experimental evidence to elucidate the mechanisms of action of ARNIs and SGLT2is and their role in chronic RV dysfunction.

## 2. Differences between RV and LV in HF

The RV and LV exhibit significant differences, including in their morphological features, biomechanics, adaptation, cellular signaling, and calcium handling. These differences may arise from their distinct origins during early development [11]. The RV arises from the secondary heart field, while the LV originates from the primary heart field [12]. These distinct origins likely influence the orientation of myocardial fibers, the fibrous continuity between the inlet and outlet, and the responses to volume and pressure overload [13]. There are also molecular differences between the RV and LV. The gene expression varies between the RV and LV in response to pressure loading and failure, such as the increase in miRNA 28, 148a, and 93 expressions in the RV, while they decrease in the LV [14]. The RV and LV exhibit distinct responses to α1 adrenergic receptor agonists and phosphodiesterase-5 (PDE5) inhibitors [15,16]. The RV shows the stronger fibrotic response to volume loading, while the LV shows a weaker response [17].

## 3. Pressure–Volume (PV) Relationship between RV and LV in HF

**Figure 1:**
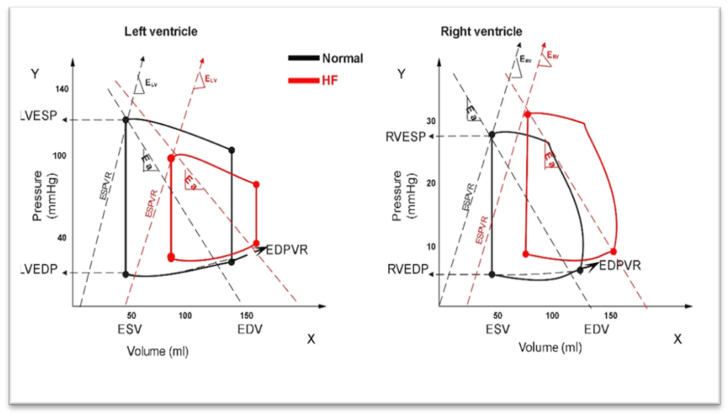
A conceptual representation of the pathophysiological pressure–volume (PV) loop. The left ventricle (LV) PV loop in normal hemodynamic condition (in black), with a rectangular PV loop, illustrating distinct isovolumetric relaxation and contraction phases, and the change in HF (in red). Right ventricle (RV) PV loop in normal hemodynamic condition (in black), with a triangular shape due to the absence of isovolumetric relaxation or contraction phases, and the change in HF (in red) (Figure represents original artwork). (ESP: end-systolic pressure; EDP: end-diastolic pressure; ESV: end-systolic volume; EDV: end-diastolic volume; ESPVR: end-systolic pressure volume relationship; EDPVR: end-diastolic pressure volume relationship; E_LV_: effective LV elastance; E_RV_: effective RV elastance; and Ea: effective arterial elastance).

Both the RV and LV share some features in their PV loops, but they differ in their shape, compliance, and response to loading states (Figure 1). The RV loop is triangular, lacking an isovolumetric relaxation phase, with output starting immediately during the systole. Understanding the PV relationship differences is crucial for tailored therapeutic approaches and improved patient outcomes. In HF, the LV–PV loop shifts to the right compared to the normal condition due to an increased LV end-diastolic volume (LVEDV) and end-systolic volume (LVESV), resulting in a decreased LV stroke volume (LVSV), end-systolic pressure (LVESP), and stroke work (LVSW). In HF, the RV–PV loop also shifts right due to an increased RV diastolic volume (RVEDV) and systolic volume (RVESV). Therefore, when exposed to identical volume overload, the RV response is larger compared to the LV, which may be caused by concomitant pulmonary hypertension (PH) [18]. When there is a chronically increased afterload, the RV pressure–volume relation shifts from a trapezoidal shape, reflecting its high efficiency/low impedance, to a square or rectangular shape, with well-developed isovolumic contraction and relaxation periods, indistinguishable from the normal LV pressure–volume loop.

## 4. RV Dysfunction with HF and Preserved Ejection Fraction (HFpEF)

In HFpEF patients, PH and RV dysfunction represent severe hemodynamic challenges [19,20]. It remains unclear whether RV dysfunction directly results from an elevated afterload or if it occurs independently of pulmonary artery systolic pressure (PASP). In an analysis of 1663 patients with HF, the patients with HFpEF, PASP > 40 mm Hg was associated with a reduced tricuspid plane systolic excursion (TAPSE) [21]. The proposed mechanisms for RV dysfunction are similar to LV dysfunction and result from autonomic nervous system activation, ischemia due to microvascular abnormality, and/or capillary rarefaction [22]. The PARAGON-HF [23] trial revealed that ARNIs did not significantly decrease mortality in HFpEF patients compared to Valsartan. However, it did reduce hospitalizations and N-terminal B-type natriuretic peptide (NT-proBNP) levels. The factors influencing RV dysfunction in HFpEF identified in the trial included a history of atrial fibrillation and flutter, LV remodeling and systolic impairment, and elevated NT-proBNP levels [24]. Notably, gender-specific differences suggest distinct mechanisms in the development of RV dysfunction in HFpEF. Gene expression profiles vary between the LV and RV and the type of LV dysfunction [25]. RV function and RV-PA coupling correlate with pulmonary congestion [26]. A small retrospective study demonstrated that ARNIs improve PH in HFpEF patients [27]. In the EMPEROR-Preserved trial, empagliflozin significantly reduced the primary endpoint of cardiovascular death or hospitalization for heart failure by 29% compared to a placebo, regardless of the presence or absence of diabetes [28,29].

## 5. RV Dysfunction with HF and Reduced Ejection Fraction (HFrEF)

The prevalence of RV dysfunction is up to 50% in patients with HFrEF, and the severity of RV dysfunction increases in proportion to the severity of LV dysfunction and is not related to PASP [30,31]. Furthermore, the prevalence of RV dysfunction, which results from LV dysfunction and is unaffected by LV ventricular assist device (LVAD) metrics, is up to 40% after continuous use of a LVAD in patients with HFrEF [32]. In these patients, survival is significantly worse among individuals with RV dysfunction than those without RV dysfunction [33]. In general, RV dysfunction and dilatation are significant predictors of adverse outcomes in patients with HFrEF [7,8,34]. RV dysfunction indicates HF progression, while its recovery is associated with an improved prognosis [35]. In an analysis of 1663 patients with HF, among those with HFrEF, a non-sinus rhythm, a high heart rate, ischemic etiology, and an E-wave deceleration time <140 ms were associated with a reduced TAPSE [21]. Observational studies have shown that ARNIs improve RV function and PH in HFrEF patients, independent of LV reverse remodeling [19,26]. Another retrospective study confirmed ARNIs’ efficacy in enhancing RV function, irrespective of demographic factors and LV metrics [36]. Moreover, adding SGLT2is, such as Dapagliflozin or Empagliflozin, to combination therapy has been associated with improvements in RV systolic function within 3–9 months [37,38,39,40]. The current approach to worsening RV failure includes a multifaceted and patient-centric approach to address the complex HF syndrome [41].

## 6. Mechanism of Action of ARNIs on RV Function

The precise mechanisms by which ARNIs benefit RV function have yet to be fully understood. Valsartan, the ARB component of ARNIs, inhibits the angiotensin II type 2 receptor (AT2R), promoting vasodilation and diuresis. Sacubitril, the neprilysin inhibitor component, is metabolized to LBQ657, which inactivates various vasoactive peptides, including natriuretic peptides (NPs), prostaglandins, bradykinin, adrenomedullin, and apelin, thereby reactivating the NO–pGC–cGMP PKG signaling pathway, which induces natriuresis, diuresis, the suppression of the renin–angiotensin–aldosterone system, and antifibrotic and anti-inflammatory actions [42]. The coadministration of Valsartan with Sacubitril can increase angiotensin levels, as angiotensin II is also a neprilysin substrate. While the specific substrates responsible for ARNIs’ benefits are unclear, studies suggest a synergistic effect of ARNIs over ACEi [43,44] and ARB [45] alone, enhancing the levels of B-type natriuretic peptide (BNP) and urinary cyclic guanosine monophosphate (cGMP) [46,47]. These changes may yield hemodynamic and biological advantages, leading to a decrease in the N-terminal brain natriuretic peptide (NT-proBNP) levels, a reliable marker of HF severity. Notably, ARNIs and BNPs are expected to increase but NT-proBNP levels decreases. Neprilysin exhibits a higher affinity for atrial-type natriuretic peptide (ANP) than BNP, resulting in a more consistent and robust increase in ANP levels [46,48].

**Figure 2:**
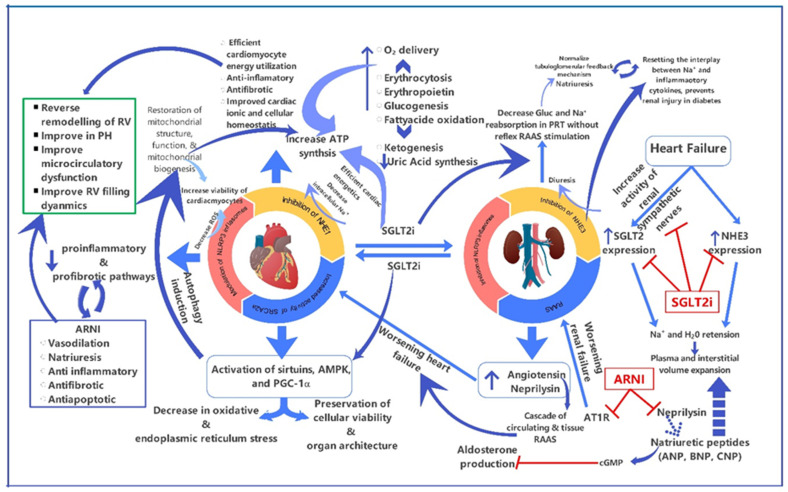
Mechanism of action of combined ARNI and SGLT2i therapy in chronic RV Failure (Figure is original artwork). (NHE1: Sodium-hydrogen exchange isoform 1; NHE3: Sodium-hydrogen exchange isoform 3; PH: Pulmonary hypertension; BNP: B-type natriuretic peptide; CNP: C-type natriuretic peptide; cGMP: cyclic guanosine monophosphate; Na+: Sodium; Gluc: Glucose; H_2_O: water; R-A-S: Renin-angiotensin-aldosterone; AT1R: Angiotensin receptor 1; ATP: angiotensin receptor 1 antagonist; ATP: adenosine triphosphate; AMPK: adenosine monophosphate kinase; PGC1∝: Peroxisome proliferator-activated receptor Ÿ 1-œ; and ROS: reactive oxygen species).

Neprilysin has a higher affinity for atrial-type natriuretic peptide (ANP) than BNP; the ANP level is increased more consistently and robustly [39]. It may be that ANP or, indeed, other neprilysin substrates (e.g., C-type natriuretic peptide, urodilatin, bradykinin, adrenomedullin, substance P, vasointestinal peptides, calcitonin gene-related peptide, glucagon-like peptide-1, and apelin) plays a predominant role in the mechanism of action of ARNIs [49,50,51]. The ANP significantly decreases sodium reabsorption in the proximal tubule by activating the NPR-A/cGMP/PKG pathway, thus increasing sodium delivery to the distal renal tubules and natriuresis [52]. ARNIs have been shown to improve in bi-ventricular failure, and experimental models of RV dysfunction due to PH [36,40,53,54,55]. Moreover, ARNIs may cause reverse remodeling of the RV by its properties of being antiapoptotic, antifibrotic, anti-inflammatory, and antithrombotic, as well as its reendothelization and modulation of gene expression, such as transferring growth factor β1 [56,57] (Figure 2).

Certain natriuretic peptides and transcription factors were upregulated in the LV, which could be linked to the mechanisms of action of ARNIs. These findings suggest that targeting these pathways has differential effects on the RV. Emerging biomarkers for RV maladaptation in pulmonary hypertension are long non-coding RNA H19, SPARC-like protein 1 (SPARCL1), and cartilage intermediate layer protein 1 (CILP1) [58]. These biomarkers can provide valuable insights into the state of the RV, especially in the context of pulmonary hypertension and other conditions that may lead to RV failure. It is important to note that while some biomarkers may offer RV-specific information, a comprehensive assessment often requires a combination of clinical evaluation, imaging, and biomarker analysis.

## 7. Mechanism of Action of SGLT2is on RV Function

Sodium-glucose cotransporters (SGLT) are transmembrane proteins that facilitate sodium and glucose transport across cell membranes. The two most well-known members of the SGLT family are SGLT1 and SGLT2, members of the solute carrier 5A (SLC5A) gene family [59]. SGLT2, primarily found in renal epithelial cells and occasionally in cardiac myocytes, especially under hyperglycemic conditions [60], plays a significant role in these processes. SGLT2 inhibitors (SGLT2is), such as Dapagliflozin and Empagliflozin, exhibit a higher affinity for SGLT2 over SGLT1, influencing their therapeutic effects [61].

SGLT2is’ direct action on cardiac myocytes involve modulating the nutrient signaling pathways, leading to the increased activity of enzymes like AMPK and SIRT1, which promote cellular health and energy efficiency [62]. This results in reduced oxidative stress, endoplasmic reticulum stress, the restoration of mitochondrial health, enhanced mitochondrial biogenesis, decreased proinflammatory and profibrotic pathways, and ultimately, preserved cardiac and renal integrity (Figure 1). SGLT-2i promotes sodium and glucose excretion by inhibiting SGLT2 in the proximal renal tubule and activates Na+/K+ ATPase of the epithelial tubular cell in the kidney, leading to natriuresis and glucosuria [63]. SGLT2is differ from traditional diuretics because it does not activate the neurohormonal axis or renal sympathetic nervous system, which can exacerbate LV remodeling and worsen HF [64].

Moreover, SGLT2is also have anti-inflammatory effects and decrease meta-inflammation [65], which is described as a slow chronic inflammatory state that leads to systemic microvascular changes that lead to widespread endothelial dysfunction in cardiac myocytes by suppressing the secretion of ICAM-1, VCAM-1, TNF-α, IL-β, and IL-6 [66].

Furthermore, SGLT2is block the sodium/hydrogen exchanger 1 cotransporter at the cardiomyocyte level, triggering a signaling cascade that decreases myofilament stiffness, hypertrophy, fibrosis, and free radical production, thus protecting cardiac myocytes [67,68,69]. Their anti-inflammatory properties further contribute to reducing systemic microvascular changes and endothelial dysfunction [59].

Clinical trials have underscored the importance of SGLT2is in managing HF and chronic renal disease, irrespective of diabetes status [70,71]. Empagliflozin reduced PAP, and the reduction was amplified over time, independent of diuretics in a randomized control trial [72].

Potential biomarkers are useful in the diagnosis and prognosis of RV dysfunction in HF, and these biomarkers can offer future novel therapeutic avenues for improving the RV function alongside improving the LV [25,54]. Furthermore, the study by Frisk et al. [73] provides valuable insights into the transcriptomic differences between the LV and RV in patients with varying degrees of HF.

MicroRNAs (miRNAs) and Insulin-like Growth Factor (IGF) are two emerging biomarkers that have shown the potential to help with HF diagnosis and prognosis. Ongoing research has unearthed various potential biomolecules, including cardiac troponins (cTn), galectin-3, soluble Suppression of Tumorigenesis-2 (ST2), and growth differentiation factor 15 [74].

These biomarkers can be used alongside traditional measures to provide a more comprehensive assessment of HF, particularly in RV dysfunction. They offer a window into the underlying pathophysiological processes and could guide more personalized treatment strategies. There are several ongoing trials, and the trials are looking at the following five major issues targeting RV failure alongside LV failure (Table 1).

Furthermore, the potential roles of genetic and epigenetic factors in RV failure are complex and multifaceted. Here is a summary of the current understanding.

**Genetic Factors**: The RV and left ventricle (LV) have different embryologic origins, likely influencing their chamber-specific responses to physiological and pathological stress [79]. The variations in the genes involved in hypoxia signaling, metabolic regulation, and neurohormonal regulation can affect the RV’s response to hypoxia, pressure or volume overload, and surgical injury [17]. There is evidence of a recapitulation of the fetal gene pattern in dysfunctional or failing RV myocardium, with changes in the expression of myosin heavy chain genes [14].

**Epigenetic Factors**: DNA methylations and DNA methyl transferase (DNMT) are crucial in regulating angiogenesis, which is essential for RV function. Aberrant methylation patterns can lead to suppressed angiogenesis and contribute to RV failure. Specific microRNAs, such as miR-126, are important regulators of angiogenesis [14]. Decreased levels of miR-126 have been associated with reduced microvessel density in the RV, contributing to its failure. Histone modifications can also affect the gene expression related to RV development and function [13].

These genetic and epigenetic factors contribute to the pathogenesis of RV failure by affecting the cellular and molecular pathways that regulate the structure and function of the RV. Ongoing research continues to unravel the complexities of these contributions, which may lead to novel therapeutic approaches for RV failure.

## 8. Role of ARNIs or SGLT2is in Primary RV Dysfunction

The specific mechanisms of ARNIs and SGLT2is in RV cardiomyopathies, such as Arrhythmogenic RV Cardiomyopathy (ARVC), still need to be fully established. ARVC, characterized by impaired desmosome function, often leads to decreased RV contractility and failure as arrhythmias progress [80]. Notably, Matiz et al. reported symptomatic and imaging-proven RV recovery in an ARVC patient treated with ARNIs [81]. Emerging evidence suggests ARNIs’ potential antiarrhythmic effects in clinical and experimental settings [82]. Additionally, Dapagliflozin has shown promise in attenuating cardiac fibrosis and inflammation, potentially benefiting RV function indirectly [83]. Dapagliflozin reduces the vulnerability to PH-induced RV dysfunction by restoring calcium handling in rat models [84,85]. Studies have also indicated SGLT2is’ role in lowering arrhythmia risks, particularly in HFrEF patients, with the long-term follow-up showing significant improvements [86]. Many studies show the benefits of SGLT2i in transthyretin and diabetic cardiomyopathies [87,88,89,90].

## 9. Role of ARNIs or SGLT2is in RV Dysfunction Due to Volume Overload

The distinction between RV dysfunction due to volume versus pressure overload has yet to be fully understood. By dilating, the RV adapts to volume overload, such as tricuspid regurgitation, but chronic overload can lead to dysfunction [91]. While direct studies on ARNIs and SGLT2is’ impact on volume overload are scarce, ARNIs’ dual inhibition of neprilysin and AT2R may benefit RV function in HF patients with volume overload [92]. For conditions like tetralogy of Fallot and Ebstein’s anomaly, ARNIs could help preserve the size and function of the RV [93,94]. Observational studies suggest a link between ARNIs and RV dilatation and function improvements in HFrEF patients [36,40,55,95,96]. Similarly, SGLT2is have improved RV dysfunction, independent of LV remodeling [38,39,97]. Experimental models and small clinical trials hint at the potential benefits of SGLT2is on RV function, paving the way for future research in this area [98,99,100].

## 10. Role of ARNIs or SGLT2is in RV Dysfunction Due to Pressure Overload

RV dysfunction due to pressure overload, such as in PH, prompts an initial adaptive response of increased contractility and hypertrophy [91]. However, this compensatory mechanism often becomes inadequate as the pressure load escalates, leading to RV failure—the end-stage manifestation of PH [6]. Traditional pulmonary vasodilators are the cornerstone of therapy, particularly for Group 1 PH patients, as they alleviate RV afterload. Yet, not all patients, especially those with PH and HFrEF, benefit optimally from drugs like prostaglandins, endothelin receptor antagonists, or phosphodiesterase inhibitors [101].

The 2015 AHA consensus statement was conservative, recommending ARNIs and other neurohormonal therapies only for RV failure secondary to PH or left heart disease if accompanied by hypertension or coronary artery disease [91]. However, emerging evidence suggests a shift in this paradigm. Recent trials have demonstrated that SGLT2 inhibitors, when combined with standard HF medications, enhance RV function more effectively than PH drugs alone [38,39,102,103,104,105]. This is corroborated by studies indicating that adding SGLT2is to PH therapy improves RV function [105,106].

Experimental research, such as the work by Connelly et al. [107], has shown that Dapagliflozin mitigates the structural, functional, and molecular effects of RV pressure overload, evidenced by reduced remodeling in PA banding-induced models or monocrotaline (MCT)-treated PH in rats [108]. Similarly, ARNIs have been observed to decrease PH, reverse vascular remodeling, and reduce RV hypertrophy and dilatation in preclinical rat studies, suggesting its potential utility in treating PH and RV dysfunction [109,110,111].

Whether early intervention with these therapies can prevent RV failure as a result of a progression in pressure and volume overload, ongoing research and future studies will be pivotal in the answer and in shaping the therapeutic approach to RV dysfunction due to pressure overload.

## 11. Role of ARNIs or SGLT2is in Systemic RV Dysfunction

Systemic right ventricle (sRV) dysfunction, as seen in transposition of the great arteries (d-TGA) after the Senning or Mustard procedures, the single RV Fontan procedure, and congenitally corrected transposition of the great arteries (ccTGA), poses a high risk of maladaptation to the systemic cardiac output. This can lead to progressive enlargement, dysfunction, and worsening tricuspid regurgitation (TR), along with atrial and ventricular arrhythmias [112,113]. The gene expression in the sRV differs due to its unique functional demands, affecting contractile proteins, ion channels, and remodeling factor [12]. The mechanisms contributing to sRV dysfunction include a compromised coronary flow reserve, perfusion defects, myocardial fibrosis, TR, mechanical dyssynchrony, and arrhythmias [114]. Although ARNIs and SGLT2is are not standard treatments for sRV, their cardiovascular benefits could improve the function and prevent myocardial damage [115]. Studies have shown that ARNIs [116,117,118,119,120,121,122,123,124] and SGLT2is [99,125,126,127,128,129,130,131] have positive effects on cardiac remodeling, inflammation, and fibrosis in both pediatric and adult patients with congenital heart disease (ACHD) and sRV or single RV Fontan. The impact of quadruple combination therapy on reverse remodeling and sRV failure warrants further investigation.

## 12. Role of Combined ARNI and SGLT2i Therapy in RV Dysfunction

The mechanism of action of the combined use of ARNIs and SGLT2is in HFrEF or HFpEF has been synergistic and promising for bi-ventricular failure (Figure 2). Meta-analyses have highlighted the superiority of this combination over individual therapies in reducing cardiovascular death and all-cause mortality, albeit with an increased risk of volume depletion [132,133]. Direct comparison trials are needed to determine if one therapy significantly outperforms the other in reducing HF risk and preserving RV function. Some evidence suggests a preference for SGLT2is due to their renal protective benefits in diabetic patients, with effects on myocardial infarction, ischemic stroke, and heart remodeling comparable to ARNIs [134]. A meta-analysis indicates that a combination of SGLT2is, ARNIs, BBs, and MRAs is highly effective in reducing cardiovascular outcomes in HFrEF patients [135]. Such combination therapy could improve the RV function in both HFrEF and HFpEF patients. However, high medication costs and limited evidence often limit the simultaneous initiation of both drugs.

The treatment landscape for HF is rapidly evolving, with ongoing research and clinical trials heralding a new era of innovative therapies. Looking ahead, a plethora of promising pharmacological agents are on the horizon for treating aHFrEF in adults. Currently, there are 119 trials underway exploring treatments for HF. A suite of novel drugs at the forefront of HF therapy is emerging. Yet, their role in RV failure remains to be explored, primarily due to a shortage of clinical trials that focus specifically on RV failure. Among these potential new treatments are Omecamtiv mecarbil, Serelaxin, Istaroxime, Ularitide, Elamipretide, Finerenone, APD418 (a β3-Adrenergic Receptor Antagonist), IONIS-AGT-LRx, and JK07, among others. As the field advances, these agents are poised to revolutionize the management of RV failure in adults. Notably, Vericiguat and Omecamtiv have been utilized based on clinical experience and consensus statements for treating RV failure.

**Limitations** of current GDMT for HF to treat RV failure: The current GDMT [1,2] for HF primarily focuses on LV dysfunction, and there are several drawbacks when applying these guidelines to treat RV failure. There is a lack of clinical trials specifically targeting RV failure [41]. This means that the efficacy and safety of these therapies for RV failure need to be well established. RV failure has a different pathophysiology compared to LV failure. For instance, RV failure is often associated with PH, which is not the primary concern in LV failure [58]. Therefore, the treatments that are effective for LV failure may not be directly applicable or beneficial for RV failure. Certain medications used in GDMT, such as beta-blockers, can worsen RV function due to their negative inotropic effects, especially in patients with pre-existing RV dysfunction. The patients with significant RV dysfunction are often underrepresented in HF clinical trials, leading to a gap in knowledge about the optimal management of RV failure. RV failure usually requires a more complex management strategy that addresses the underlying cause, such as PH. It may involve targeted therapies that are not part of the standard GDMT [41]. The biomarkers and clinical endpoints used to monitor LV failure may be less effective for monitoring RV failure, necessitating the development of RV-specific markers and endpoints [74]. These drawbacks highlight the need for more RV-specific research and new guidelines that address the unique aspects of RV failure management.

**Future directions**: The future direction for the treatment of RV failure is multifaceted, focusing on medical and mechanical interventions tailored to the unique physiology of the RV [41]. It is important to enhance the timely identification of RV failure through advanced diagnostic tools like magnetic resonance and radionuclide imaging. These tools are crucial for assessing RV function and guiding treatment. The medical treatment of RV failure typically involves two approaches: perfusion pressure restoration and myocardial contractility improvement [76]. Future therapies may include novel pharmacological agents targeting the molecular pathways involved in RV dysfunction [73]. The use of mechanical assist devices, such as mechanical circulatory support (MCS), is being explored for managing acute RV failure, especially where there is insufficient pharmacotherapy. These devices can improve hemodynamics and support a failing RV [75,77]. In cases where recovery is not observed with medical management or mechanical support, heart transplantation remains a definitive treatment option. Future strategies may improve the selection and management of candidates for transplantation.

## 13. Conclusions

This review underscores the complex interplay of ventricular function within the HF continuum. An RV failure when it occurs alongside an LV failure, whether the RV failure is a separate entity or due to an LV failure, requires further clarification. Pending LV- or RV-specific therapies, the early initiation of ARNIs and SGLT2is in line with GDMT for eligible HFrEF and HFpEF patients is vital to address RV dysfunction. Large-scale, multicenter randomized controlled trials are imperative to ascertain the efficacy and safety of ARNIs/SGLT2is and to determine whether an improvement in the RV function is independent of the LV function enhancement. RV dysfunction merits a focus in future HF clinical trials, representing a significant gap in current management strategies. Incorporating the RV function into risk stratification and trial endpoints could optimize patient outcomes, particularly in selecting HFrEF candidates for LVAD and tailoring individualized drug therapies.

## Figures and Tables

**Table 1:** Summarizes the goals of ongoing trials.

Authors	Study	What the Study Entails
Salam et al. [75]	RV Shock Management	Strategies for managing RV shock, particularly in the setting of RV infarct
Mansoor et al. [76]	Pharmacotherapy for HF	Trials looking at novel medications, as well as currently approved drugs
Yuriditsky et al. [77]	Medical Optimization of Acute RV Failure	Tailored volume administration, ideal vasopressor selection, inotropes to restore contractility, and pulmonary vasodilators to improve afterload
Diaz JC et al. [78]	Left Bundle Branch Area Pacing (LBBAP)	Investigation of LBBAP in the context of RV depolarization provided by the right bundle branch
Lippman et al. [79]	Pathogenesis of RV Dysfunction	New insights into the role of fibrosis, inflammation, myocyte contraction, and mitochondrial dynamics in RV dysfunction
